# Editorial: Transcriptional and post-transcriptional regulatory networks in cellular homeostasis and stress response

**DOI:** 10.3389/fmolb.2026.1844239

**Published:** 2026-04-27

**Authors:** Alessandra Boccaccini, Davide Capauto, Matteo Cassandri, Silvia Santopolo

**Affiliations:** 1 Department of Science and Bio-Technology, Università Campus Bio-Medico di Roma, Rome, Italy; 2 Department of Psychiatry, Division of Molecular Psychiatry, Yale University School of Medicine, New Haven, United States; 3 Department of Experimental Medicine, Sapienza University of Rome, Rome, Italy; 4 Innate Lymphoid cells Research Unit, Bambino Gesù Children’s Hospital, IRCCS, Rome, Italy

**Keywords:** homeostasis, post-transcriptional control, RNA, stress response, transcriptional regulation

Cells rely on the dynamic regulation of homeostasis to ensure their survival, maintain the integrity of essential physiological processes, and preserve the stability of the intracellular environment. This finely balanced state, however, is constantly challenged by external perturbations, including environmental fluctuations, nutrient availability, oxidative stress and other cellular insults. Consequently, the ability to promptly restore homeostasis following perturbation is critical, as its failure can lead to functional impairment, accumulation of damage, and cell death or disease.

To achieve this, cells developed sophisticated regulatory mechanisms operating at multiple levels, including both transcriptional and post-transcriptional control. These systems enable all organisms to sense perturbations, rapidly adapt gene expression programs and fine-tune protein synthesis, ensuring preservation of cellular functions and adaptive responses to fluctuating internal and external conditions (Kü and ltz, 2005).

Barańska et al. highlighted the central role of transcriptional control in homeostasis regulation in prokaryotes, which represent relatively simple cellular systems. In these organisms, second messengers, such as cAMP, coordinate the expression of numerous genes in response to environmental changes. This regulation is further shaped by chromosomal organization, since DNA topology, modulated by enzymes like topoisomerases, determines the accessibility of genomic regions, influencing global transcription. Prokaryotic cells can rapidly adapt to nutrient fluctuations through transcriptional regulations, such as iron homeostasis mediated by regulators like Fur. Similarly, defense mechanisms, including toxin-antitoxin systems and anti-phage responses, are transcriptionally coordinated to ensure rapid protection against environmental threats. Beyond these single-cell responses, transcriptional reprogramming also underlies the formation of biofilms, allowing cells to transition into multicellular-like communities that exhibit enhanced resistance to environmental challenges.

Gene regulatory mechanisms have diversified throughout evolution, and in eukaryotes, regulatory complexity relies on specialized transcriptional elements, including super-enhancers (SEs), which play key roles in maintaining cellular identity and homeostasis. Wang et al. described SEs as large clusters of enhancer sequences with exceptionally strong transcriptional activation ability that promote high expression levels of target genes through dense occupancy of cell type-specific transcription factors and cofactors, linking transcriptional and post-transcriptional control. A key feature of SEs is indeed the production of enhancer RNAs (eRNAs), non-coding transcripts which subsequently fine-tune gene expression through RNA-mediated mechanisms. Acting as integrative platforms, SEs respond to developmental and environmental cues, ensuring precise spatiotemporal regulation of transcription. This is particularly evident in cancer, where SEs drive oncogene expression (e.g., c-MYC), or are newly formed, as observed in T-cell acute lymphoblastic leukemia (T-ALL) with TAL1 overexpression. SEs also regulate tumor-specific transcripts and represent promising therapeutic targets. Beyond cancer, SE-associated regions are enriched in disease-linked genetic variants, highlighting their importance in broader pathological contexts, including autoimmune and neurodegenerative diseases.

The exposure to certain stimuli induces a dynamic regulation of transcription, which can modulate cellular responses to stress. Among these stress factors, High-Power Microwaves (HPM) are becoming increasingly relevant as their applications continue to expand. This raises important questions about potential biological effects and safety. In this regard, Gao et al. observed that exposure of corneal epithelial cells to HPM at 4,3 GHz induces a range of significant cellular changes: elevated reactive oxygen species, collapse of mitochondrial membrane potential, high apoptosis rates, and sustained inhibition of proliferation. Notably, these effects appear to be transcriptionally mediated, as shown by suppression of the mTOR signaling pathway, upregulation of TSC2, and activation of Polycomb-mediated chromatin remodeling.

Another strategy by which eukaryotic cells respond to stress is the formation of biomolecular condensates through liquid-liquid phase separation, a process in which proteins and RNAs dynamically and reversibly segregate from the cytoplasm into functional assemblies.

Among these, stress granules (SGs) are transient membraneless structures that repress mRNA translation to preserve cellular homeostasis. Zhang et al. highlighted their key role in liver diseases, particularly hepatocellular carcinoma, where multiple SG-related genes, including *DDX1*, *DKC1*, *BICC1*, are dysregulated with a four-genes signature that predicts prognosis. SGs contribute to therapy resistance, as sequestration of PTEN mRNA promotes tumor progression and tyrosine kinase inhibitors resistance. Also, during hepatitis C virus (HCV) infection, PKR and eIF2α phosphorylation induces SGs formation, suppressing antiviral gene translation and enhancing viral replication, while the combination of HCV infection and IFNα induces SGs changes which lead to the establishment of persistent infection. Finally, in fatty liver disease, loss of core SG proteins, including G3BP1, TIA1, DDX3X, worsens pathology, underscoring their protective function.

Arsiè et al. pointed out that RNA modifications, notably N6-methyladenosine (m6A) and N1-methyladenosine (m1A), are key regulators of biomolecular condensates, influencing their formation, stability, and dynamics. Functionally, m6A promotes SGs assembly, while m1A protects RNAs by altering local structure and protein interactions, promoting their sequestration into SGs and enabling translation recovery after stress. As RNA modifications are key regulators of biomolecular condensates, affecting both normal granule formation and pathological aggregation, reliable detection and analysis tools are essential. Most approaches use antibodies against m6A and m1A combined with sequencing of enriched RNAs. Moreover, certain modifications generate characteristic enzymatic signatures, which can be exploited for mapping. Bioinformatics tools, such as exomePeak and MACS2, identify enrichment peaks, while computational models help to discriminate true modification sites from noise and predict *de novo* methylation events.

Finally, Ou et al. focused on post-transcriptional, age-related regulatory networks in human limbal epithelial cells, investigating how alterations in mRNA and long non-coding RNA expression contribute to stress responses and cellular homeostasis. By integrating RNA sequencing with computational analyses, the authors identified age-dependent alterations in both mRNA and long non-coding RNA expression, uncovering a stress-responsive competing endogenous RNA (ceRNA) network involving SDHAP2, miR-17-5p, miR-20b-5p, and RAB11FIP1.

Collectively, this Research Topic highlights multiple layers of transcriptional and post-transcriptional regulation of cellular homeostasis and stress responses, from prokaryotes to complex eukaryotes and disease contexts. In prokaryotes, understanding these mechanisms sheds light on antibiotic resistance and synthetic biology approaches. In eukaryotes, SEs and RNA modifications emerge as central regulators, with SGs modulating cellular adaptation. Despite early-stage development, targeting SGs, RNA modifications or SEs holds therapeutic potential for liver diseases, neurodegeneration, and cancer. Future strategies, including high-throughput screening and rational design of small molecules may enable precision modulation of these regulatory networks.
